# The Curing Coma Campaign: Framing Initial Scientific Challenges—Proceedings of the First Curing Coma Campaign Scientific Advisory Council Meeting

**DOI:** 10.1007/s12028-020-01028-9

**Published:** 2020-06-23

**Authors:** J. Javier Provencio, J. Claude Hemphill, Jan Claassen, Brian L. Edlow, Raimund Helbok, Paul M. Vespa, Michael N. Diringer, Len Polizzotto, Lori Shutter, Jose I. Suarez, Robert D. Stevens, Daniel F. Hanley, Yama Akbari, Thomas P. Bleck, Melanie Boly, Brandon Foreman, Joseph T. Giacino, Jed A. Hartings, Theresa Human, Daniel Kondziella, Geoffrey S. F. Ling, Stephan A. Mayer, Molly McNett, David K. Menon, Geert Meyfroidt, Martin M. Monti, Soojin Park, Nader Pouratian, Louis Puybasset, Benjamin Rohaut, Eric S. Rosenthal, Nicholas D. Schiff, Tarek Sharshar, Amy Wagner, John Whyte, DaiWai M. Olson, the Neurocritical Care Society Curing Coma Campaign

**Affiliations:** 1 Department of Neurology and Neuroscience, University of Virginia, Charlottesville, VA USA; 2 Department of Neurology, Zuckerberg San Francisco General Hospital, University of California, San Francisco, Building 1, Room 101, 1001 Potrero Avenue, San Francisco, CA 94110 USA; 3 Department of Neurology, Columbia University Irving Medical Center/New York Presbyterian Hospital, New York, NY USA; 4 Department of Neurology, Massachusetts General Hospital, Harvard Medical School, Boston, MA USA; 5 Department of Neurology, Neurocritical Care, Medical University of Innsbruck, Innsbruck, Austria; 6 Departments of Neurology and Neurosurgery, David Geffen School of Medicine at UCLA, Los Angeles, CA USA; 7 Department of Neurology, Washington University, Barnes-Jewish Hospital, St Louis, MO USA; 8 Department of Biomedical Engineering, Worcester Polytechnic Institute, Worcester, MA USA; 9 Departments of Critical Care Medicine, Neurology, and Neurosurgery, University of Pittsburgh/UPMC Health System, Pittsburgh, PA USA; 10 Departments of Anesthesiology and Critical Care Medicine, Neurology and Neurosurgery, Johns Hopkins University School of Medicine, Baltimore, MD USA; 11 Division of Brain Injury Outcomes, Johns Hopkins University, Baltimore, MD USA; 12 Departments of Neurology, Neurosurgery and the Beckman Laser Institute, University of California-Irvine, Irvine, CA USA; 13 Department of Neurology, Feinberg School of Medicine, Northwestern University, Chicago, IL USA; 14 Department of Neurology, University of Wisconsin-Madison, Madison, WI USA; 15 Department of Neurology and Rehabilitation Medicine, University of Cincinnati Gardner Neuroscience Institute, University of Cincinnati College of Medicine, Cincinnati, OH USA; 16 Department of Physical Medicine and Rehabilitation, Spaulding Rehabilitation Hospital, Harvard Medical School, Boston, MA USA; 17 Department of Neurosurgery, University of Cincinnati College of Medicine, Cincinnati, OH USA; 18 Departments of Neurology and Neurosurgery, Washington University, Barnes-Jewish Hospital, St Louis, MO USA; 19 Department of Neurology, Rigshospitalet, Copenhagen University Hospital, Copenhagen, Denmark; 20 Department of Neurology, The Johns Hopkins University School of Medicine, Baltimore, MD USA; 21 Departments of Neurology and Neurosurgery, New York Medical College, Valhalla, NY USA; 22 College of Nursing, The Ohio State University, Columbus, OH USA; 23 Division of Anaesthesia, University of Cambridge, Cambridge, UK; 24 Department and Laboratory of Intensive Care Medicine, University Hospitals Leuven and KU Leuven, Leuven, Belgium; 25 Department of Psychology, University of California, Los Angeles, CA USA; 26 Department of Neurosurgery, David Geffen School of Medicine at UCLA, Los Angeles, CA USA; 27 Department of Anesthesiology and Critical Care, Sorbonne University, GRC 29, AP-HP, DMU DREAM, Pitié-Salpêtrière Hospital, 75013 Paris, France; 28 Department of Neurology, Neuro-ICU, Sorbonne University, Pitié-Salpêtrière Hospital, Paris, France; 29 Departments of Neurology, Neuroscience, and Medical Ethics, Weill Cornell Medicine, New York, NY USA; 30 Neuro-anesthesiology and Intensive Care Medicine, Sainte-Anne Hospital, Paris-Descartes University, Paris, France; 31 Experimental Neuropathology, Infection and Epidemiology Department, Institut Pasteur, Paris, France; 32 Department of Physical Medicine and Rehabilitation, Department of Neuroscience, Clinical and Translational Science Institute, University of Pittsburgh, Pittsburgh, PA USA; 33 Moss Rehabilitation Research Institute, Elkins Park, PA USA; 34 Department of Neurology and Neurotherapeutics, University of Texas Southwestern, Dallas, TX USA

**Keywords:** Coma, Endotype, Biomarker, Consciousness, Recovery

## Abstract

Coma and disordered consciousness are common manifestations of acute neurological conditions and are among the most pervasive and challenging aspects of treatment in neurocritical care. Gaps exist in patient assessment, outcome prognostication, and treatment directed specifically at improving consciousness and cognitive recovery. In 2019, the Neurocritical Care Society (NCS) launched the Curing Coma Campaign in order to address the “grand challenge” of improving the management of patients with coma and decreased consciousness. One of the first steps was to bring together a Scientific Advisory Council including coma scientists, neurointensivists, neurorehabilitationists, and implementation experts in order to address the current scientific landscape and begin to develop a framework on how to move forward. This manuscript describes the proceedings of the first Curing Coma Campaign Scientific Advisory Council meeting which occurred in conjunction with the NCS Annual Meeting in October 2019 in Vancouver. Specifically, three major pillars were identified which should be considered: endotyping of coma and disorders of consciousness, biomarkers, and proof-of-concept clinical trials. Each is summarized with regard to current approach, benefits to the patient, family, and clinicians, and next steps. Integration of these three pillars will be essential to the success of the Curing Coma Campaign as will expanding the “curing coma community” to ensure broad participation of clinicians, scientists, and patient advocates with the goal of identifying and implementing treatments to fundamentally improve the outcome of patients.

Coma is a clinical condition common across numerous acute neurological disorders and non-neurological disorders (such as drug overdose and decompensated metabolic disease). Coma can herald long-lasting unconsciousness, a transient state followed by return of consciousness, or a chronic state characterized by partial recovery of consciousness [1–3]. The prognosis for return of consciousness is a critical determinant of family and medical care decisions regarding goals of care; whether to aggressively attempt to support patients or implement comfort measures (with the frequent outcome of death) [4, 5]. The most common and compelling issues faced by patients, families, and clinical care providers are whether a patient with impaired consciousness ***can*** ‘wake up’; if so, what level of awareness they can be expected to recover to, what the timeline for such a recovery may be, and what will it take to get there. Uncertainty in current methods of prognostication and the lack of pharmacological, surgical, and rehabilitation interventions that can specifically improve consciousness are the most encompassing challenges currently facing neurocritical care. In addition, concern regarding a self-fulfilling prophecy of poor outcome due to withdrawal of support in patients who might otherwise have a favorable outcome extends across acute neurological conditions, with traumatic brain injury as but one example [6]. Recognizing this gap in coma understanding and intervention, and the key importance of advancing research in this area, the Neurocritical Care Society (NCS) has launched the Curing Coma Campaign [7].

By definition, coma defines a subset of patients with disorders of consciousness (DOC) and refers to a state of lack of arousal in which a brain injured patient has no interaction with his or her environment. DOC is a broader definition that encompasses patients with altered consciousness that may be less complete than coma. It should be emphasized that while clinical definitions of states of disordered consciousness have recently been rigorously addressed (e.g., coma, vegetative state/unresponsive wakefulness syndrome [VS/UWS], and minimally conscious state [MCS]), these constructs have been primarily implemented in the subacute and chronic phase of illness [3].

Acute coma and impaired consciousness have been traditionally studied and treated in a disorder-specific context. For example, cardiac arrest, traumatic brain injury, and brainstem stroke are often considered separately with regard to prognosis, intervention and rehabilitation strategies. However, the minimum common-denominator anatomy and physiology of altered consciousness across diseases might be more relevant when considering targeted interventions to improve patients with DOC [8–11]. Advancing knowledge about the pathogenesis of altered consciousness has potential to generate fundamental revelations that can be used to develop treatments for patients with a broad range of DOC etiologies [12–15].

The Curing Coma Campaign aims to “change the game” by moving beyond the limitations imposed by considering a specific phase of illness such as subacute/chronic or disorder-specific investigation in order to cover the entire disease narrative, from ictus to outcome, and articulate a comprehensive and rational clinical taxonomy of severe DOC. The campaign also aims to go beyond a simple descriptive exercise and use this classification as a basis to dissect mechanisms, improve prognostication, identify test therapies, and impact outcomes. Perhaps most importantly, we wish to use this campaign as a means of bridging the presentation of coma in the acute phase to its eventual outcome. This will necessitate engagement with clinicians in post-acute care, since a continuum approach provides the best chance of making meaningful changes that affect the lives of our patients. By bridging current consciousness science with new research regarding patients with DOC across a broad array of initial causes, the intent is to develop an enduring framework for studying, promoting awareness, and developing treatments. Furthermore, as neurocritical care expands worldwide, it is a priority to identify ways to improve care that can be implemented across differently resourced environments [16, 17].

With its launch in 2019, one of the first efforts of the Curing Coma Campaign was to bring together a diverse group of coma scientists, neurointensivists, neurorehabilitationists, and implementation experts into a Scientific Advisory Council (SAC). The initial task of the SAC was to identify scientific gaps in order to facilitate the development of a scientific “roadmap.” The Curing Coma Campaign SAC met for the first time in person during the NCS Annual Meeting in Vancouver in October 2019. This manuscript describes the overall proceedings of that first meeting, addressing three fundamental and overarching pillars to lay the foundation for next steps. These pillars are (1) *Endotyping*—developing a better understanding of the different types of coma, (2) *Biomarkers*—evaluating current tools and their shortcomings in understanding coma and its prognosis, and (3) *Proof-of-Concept Clinical Trials*—identifying early proof-of-concept interventional studies to evaluate new treatment protocols and inform clinical trial design. Three separate groups were tasked with describing the background, current understanding, and impact for patients and families for each pillar. In addition, they were asked to summarize the integral problems and to propose next steps. Principles were then reviewed, discussed, and revised by the entire SAC.

## Endotyping

This pillar was initially considered as “phenotyping” of DOC. However, the term “phenotype” was recognized as being limited to the clinical manifestations of DOC, primarily as evidenced by the neurological examination. The fact that different disease entities and structural or metabolic mechanisms can result in similar ‘clinical phenotypes’ was identified as one of the reasons for current limitations in coma assessment and treatment. Thus, this pillar was changed to endotyping to more accurately reflect the gap and challenge faced. To date, DOC endotypes in the acute stage have not been well classified. A better identification of DOC endotypes and their recovery trajectories early in the course of intensive care unit (ICU) management may delineate patients with a high chance of recovery and distinguish those who may be amenable to specific interventions from those with a poor likelihood of recovery.

### Current Approach in DOC Classification and Clinical Recovery Trajectories

Clinicians are regularly confronted with the difficult challenges of assessing the cause of coma, prognosis for recovery, and expected trajectory of recovery. Clinical assessment typically relies on the observed behavior of the patient and results from clinical examination testing. However, clinical examination may be insensitive for the detection of recovery of consciousness and other cognitive functions, which may only be captured by novel methods such as advanced neuroimaging or electrographic studies. So far, no coma assessment battery exists that can properly and definitively endotype DOC in the ICU. This leads to uncertainty regarding prognosis and trajectory of recovery, as well as increasing the potential for misclassification. The assessment of coma, and related states such as UWS and MCS, is limited due to patient-specific factors, inconsistent interrater reliability in the clinical exam, and factors that may confound clinical assessment such as intubation and sedation [18]. Recent guidelines pertaining to DOC, focused on subacute and chronic patients, have assessed the current state of evidence and provided a framework for addressing gaps [19, 20].

Current prognostic models are deficient with regard to the time window and degree of expected recovery. Arbitrary time points for outcome assessment that may have been chosen for pragmatic reasons often fail to capture functional recovery. For example, assessment at 3 months may not capture ongoing recovery that can continue for 1 year after injury and beyond, even in patients who do not regain full consciousness in the ICU [21].

Individual patients with different trajectories are commonly lumped together, providing an incomplete view of heterogeneous recovery patterns that may be related to nuanced anatomic or functional circuitry impairment. Additionally, widespread subcellular functional alterations may endure for long periods of time producing sustained coma in brains harboring a potential for good outcomes [22]. The ultimate goal is to endotype DOC in order to achieve precision diagnosis for the cause and recovery trajectory. This would allow clinically and biologically relevant subgroups to be parsed for improved prognostication and targeted interventions.

### Proposed Classification of Different Coma Endotypes and Recovery Trajectories

An initial classification of DOC endotypes could focus on how the specific coma/DOC mechanism is linked to recovery and potential interventions. Four different general subgroups emerge:

1. *DOC endotype without commensurate structural damage* In principle, these could be reversible using traditional treatment approaches (e.g., seizures, drug overdose) and are recognized based on metabolic or electrical instability.
2. *DOC endotype with structural or functional damage that is amenable to replacement or bypass therapy* This would include damage that could potentially be replaced by surrogate brain function such as stimulant medications or brain-machine interfaces [12, 23]. Even if interventions are not present today, this endotype would represent a high-priority and fertile target for identifying and testing new interventions. Except in the most severe cases, hypoxic-ischemic injury and traumatic brain injury would fit in this category.
3. *DOC endotype that is not amenable to pharmacologic or anatomic replacement or repair therapy* Essentially, this would be untreatable conditions based on the underlying biology, such as end-stage prion or Alzheimer disease. This endotype may progress from endotype 1 or 2 when metabolic, electrical, or structural disease is not reversed. It would be hoped that this endotype might be a “moving target” in which novel therapies emerge that can arrest disease progression and move patients into endotype 1 or 2 by virtue of more successful therapies to reverse metabolic or electrical instability or provide replacement or bypass therapy.
4. *DOC mimics endotype* in which structural damage results in a syndrome that mimics or is mistaken for a DOC. Examples could include severe aphasia, abulia, or locked-in syndrome. Such endotyping could allow targeted treatment for these alternate impairments with similarly profound functional impact.

### Potential Methods to Classify Different Coma Endotypes

Multimodal tools exist to endotype coma and DOC [19, 24]. Neuroimaging methods such as magnetic resonance imaging (MRI) (e.g., resting state functional MRI, task-based functional MRI, and diffusion tensor imaging [DTI]) and positron emission tomography (PET) (e.g., resting state PET) have been used to identify patients with preserved neural networks (MRI/DTI and PET) and covert consciousness (task-based fMRI) [25, 26]. At present, it is not clear which brain circuits are required to regain consciousness and what morphological changes translate to clinical trajectories. Likewise, electrophysiologic methods (electroencephalography [EEG], high-density EEG [hdEEG], transcranial magnetic stimulation EEG [TMS-EEG]) enable refined evaluation of consciousness and higher-order cortical function [27–29]. Functional networks in the brain at rest are associated with the state of consciousness in DOC [30]. Specifically, resting default mode network connectivity may be an important concept to characterize the state of consciousness underpinned by electrophysiologic networks [31, 32]. Electrographic studies may be combined with active and passive perturbation tasks to further elucidate different endotypes of coma. Other invasive monitoring methods also provide assessment of the brain physiological state, oxidative substrate delivery (e.g., brain tissue oxygenation, regional cerebral blood flow [rCBF], intracranial pressure [ICP], cerebral microdialysis, brain water, brain temperature, spreading depolarizations), and serum and cerebrospinal fluid (CSF) metabolic biomarkers.

### Benefits to the Patient, Family, and Clinicians

Early recognition of different endotypes of coma may identify patients with various recovery trajectories early in the ICU setting, leading to improvement in the accuracy of early neuro-prognostication. Identification of patients amenable to interventions at specific time points will enable clinical trials of targeted treatments to accelerate emergence from coma and improve cognitive and functional outcome. A valid trajectory model would facilitate communication between the patient, family members, and clinicians.

### Next Steps in Endotyping Coma and Redefinition of Recovery Trajectories

Three next steps are suggested. First, there is a need to develop clinically and biologically relevant coma endotypes. Second, it is important to identify one or more endotypes amenable to treatment. Third, development of well-defined common data elements (CDE) for DOC and its recovery trajectory are necessary to standardize future work. This approach has the potential to dramatically advance care of comatose neurocritical care patients through identification of relevant coma endotypes early in the ICU and relating these endotypes to various trajectories, with the ultimate goal of applying individualized treatment strategies based on clinically and biologically stratified coma endotypes.

## Biomarkers

In today’s ICU, patients can be monitored with a suite of tools that includes physiological signals, imaging, fluid chemistries, neuro-electrical stimulus response, and clinician observations. While findings from these tools (biomarkers) provide a degree of information on the patient’s state, they remain insufficient to identify details of the endotype of comatose patients, cause of the coma, or severity of the disease, all of which are necessary to provide the most effective treatment and to identify new targeted interventions.

Additionally, as treatment is given, it is important that a patient’s disease state be accurately monitored to determine if the course of treatment should be modified. This will require data synchronization and integration though clinical informatics. While recovery trajectories have been identified following emergence from coma, there is a critical need to define coma recovery trajectories during the acute period [33]. Tools to follow patients throughout the course of disease are critically important to define coma recovery trajectories. Development of multidimensional flexible trajectory models that integrate clinical, neuroimaging, electrographic, and other biomarker data at different time points is a high priority. In addition, standardized approaches to clinical assessment, imaging, or other testing and adjusting for therapeutic interventions will be needed to develop validity for trajectory models.

### Current Biomarker Uses in Comatose Patients

While a range of tools to find biomarkers already exist, many are difficult to administer to comatose patients or do not provide the accuracy and precision necessary to pinpoint location (and severity) of the lesion or connectivity of integrated networks. As a result, there is a need for biomarker tests that are suitable for use in the ICU setting. Consciousness is a state that is elusive and not necessarily directly measurable. In the clinical context, assessing the presence of consciousness in a patient is based on pragmatic principles in which a patient can demonstrate voluntary/purposeful behaviors that can be taken to imply a state of (at least minimal) consciousness. When categorizing biomarkers, however, both the physiological signal that is measured as well as the context in which the signal is obtained (e.g., assessment at rest versus during a task) need to be clearly specified in order to evaluate the degree to which each biomarker is associated with the presence of consciousness. This is critical because there are fluctuations in the patient’s physical and neurological examination that relate to lesion locus, sleep–wake cycles, medication administration, and comorbidities such as infection and systemic organ dysfunction that may confound clear interpretation.

The three goals of biomarkers in patients with coma and DOC are to (1) make endotypic or mechanistic diagnostic determinations (see the section above), (2) to follow the progression of the comatose patient, either as natural history or in the context of an intervention, and (3) to develop multidimensional flexible trajectory models for comatose ICU patients using neuroimaging, electrophysiology and other biomarkers. The ideal biomarker in each situation is distinctly different. As described in the preceding section, for *endotypic biomarkers*, ideal tests need to have high sensitivity and specificity. Complexity of interpretation is probably a less important hurdle because the tests will likely be administered once. For *following disease progression and trajectory of recovery*, repeated measurements that are easy to interpret in real time are preferable to complex tests. For developing models, integration of complex and readily repeated measures will be necessary.

Another way to categorize biomarkers is to separate mechanistic markers that inform the underlying pathology from outcome markers that predict which patients will improve. This dichotomy may be useful to select biomarkers for use in different situations. For example, a mechanistic marker may be important in developing a clinical trial of an intervention but may be less useful to inform families about the patient’s prognosis. While an outcome marker may be useful in exactly the opposite circumstance.

There are specific unmet needs that will guide new biomarker development. First, unlike endotyping biomarkers that are rapidly advancing, biomarkers that identify and monitor progression of specific dysfunctional brain circuits are currently not available but would be of great value. Second, there is an unmet need for biomarkers that indicate real-time progression of brain injury. Successful evaluation of the natural history of disease and treatment response in early phases of injury depends on clear discrimination of transient versus permanent brain injury.

Finally, there is an early, persuasive case for exploring genetic variations that could drive the likelihood of prolonged DOC after an acute insult or affect the likelihood of recovery. While the likelihood of prolonged DOC has been addressed in genetic association studies (such as in TBI), direct extension to the specific outcome of poor emergence is difficult [34]. Genetic drivers of recovery trajectories are less well studied. However, it is biologically plausible to consider whether emergence from DOC could be influenced by genes responsible for cognitive reserve, sleep, inflammatory host response, late neurodegeneration, and neural regeneration and repair. Genetic markers could prove important for several reasons. Such variations may stratify patients in terms of risks of non-emergence—which is particularly important considering some patients may achieve late (and often unexpected) recoveries. Second, genetic biomarkers may allow us to identify new biological targets and develop new therapies.

### Approach to Solving the Problem—the Ideal Biomarker for Coma in the ICU

No biomarker today has the ability to clearly map the function of thalamocortical interactions or the reticular activating system, both of which are critical for consciousness. Table 1 provides a description of currently used biomarkers to assess and quantify consciousness. When considering biomarkers of consciousness, it is useful to assess them along the axes of biological signal and context. Biological signals assessed at rest quantify brain physiology that is necessary to maintain a conscious state. Passive perturbation tests measure biological signals (biomarkers) in response to external sensory or direct neuronal stimuli. The recorded signal allows an assessment of brain activation associated with stimulation, thereby providing a correlate to level of consciousness. Active perturbation tasks confront the patient with a task and measure the response. The most widely used behavioral assessments for consciousness in the clinical context evaluate eye opening, visual fixation and pursuit, and motor responses to command or stimulation. The current practice standard for behavioral assessment of consciousness is the Coma Recovery Scale Revised, which is superior to other clinical scales [35]. This assessment allows clinical classification into coma, VS/UWS, MCS (minus and plus), and emergence from MCS. Patients who demonstrate volitional responses during task-based EEG or functional MRI but who are unable to follow commands on behavioral assessment are labeled as having covert consciousness, or cognitive motor dissociation [36].

**Table 1:** Currently used biomarkers for the assessment of consciousness

*Brain at rest*
Structural assessment: brain MRI (FLAIR, high resolution T1, diffusion tensor imaging), Head CT
Neuronal activity: spectral analysis (ABCD classification), permutation entropy and spectral complexity and connectivity measures of the resting EEG
Functional connectivity: EEG coherence, phase-amplitude modulations, resting state functional MRI
Direct measures of brain physiology: metabolism (FDG PET), oxygen (partial brain tissue oxygenation and oxygen saturation), blood flow (MR or CT perfusion imaging, MR arterial spin labeling, Xenon CTP, invasive measure of rCBF), ICP/CPP, cerebral metabolites (MR spectroscopy, microdialysis), brain water content, brain temperature, cortical spreading depolarization
Measures reflecting injury to the brain: CSF and serum (NSE, S110Beta, GFAP, vimentin, myelin basic protein, inflammatory markers such as IgG electrophoresis)
*Passive perturbation tasks*
Sensory stimulation induced evoked potentials (SSEP, BAEP, MEP): short vs long latency
Event-related potentials: cognitive processing involved with processing of regularities (i.e., local global paradigm), processing of ERPs induced modulation of the autonomic nervous system
Transcranial magnetic stimulation with high-density EEG co-registration
Stimulus-based functional EEG or functional MRI (e.g., with language or music stimuli)
*Active perturbation tasks*
Behavioral assessment: coma recovery scale-revised, other less comprehensive clinical scales (FOUR score, Glasgow Coma Score), differential electromyographic response
Task-based functional EEG or functional MRI (e.g., commanded motor acts, motor imagery, or spatial navigation)

*BAEP* brainstem auditory evoked potential, *CPP* cerebral perfusion pressure, *CT* computed tomography, *CTP* computed tomographic perfusion, *EEG* electroencephalography, *ERP* event-related potential, *FDG PET* fluorodeoxyglucose positron emission tomography, *FLAIR* fluid-attenuated inversion recovery, * FOUR * full outline of unresponsiveness, *GFAP* glial fibrillary acidic protein, *ICP* intracranial pressure, *MEP* motor evoked potential, *MR* magnetic resonance, *MRI* magnetic resonance imaging, *NSE* neuron specific enolase, *rCBF* regional cerebral blood flow,*SSEP* somatosensory evoked potential

When prioritizing tests of biomarkers, accuracy of measurement (scientific yield) and availability in the ICU (applicability) need to be considered. Ideally, these tools would be available at the patient’s bedside and could be performed repeatedly (or automated). Currently, the highest yield and broadly available tests are behavioral assessments and resting EEG, which additionally elucidate biomarkers of reversible coma endotypes including seizures (endotype 1). It is crucial that the behavioral assessment is standardized, and this requires training of examiners. With additional standardized computational analyses, now publicly available, the EEG may generate immediately available useful measures of consciousness. Particularly the ABCD model is a promising resting EEG tool as it provides a hierarchical representation of the degree of thalamocortical disconnection [37]. Active paradigms in which the patient is asked to respond to command while monitored with EEG and passive approaches such as the “local–global violation” paradigms, which have been closely linked to the presence of consciousness, as well as specific markers computed from resting state analysis EEG are feasible to assess in an ICU setting and associated with good long-term functional outcomes [28, 38, 39]. However, utilization of these approaches requires replication, standardization, and computational resources [28]. Similarly, diffusion tensor imaging and resting state MRI provide an assessment of underlying structural and functional connectivity of the brain, respectively [40–42]. Like all imaging measures, these require transport of the patient out of the critical care setting and provide assessment at a limited number of time points. Much of the brain physiology, structural imaging, and injury measures are correlated with extent of impairment of consciousness and provide insight into underlying mechanisms but are low yield as biomarkers for consciousness. However, it should also be noted that the post-processing of neuroimaging data requires extensive expertise and constant monitoring of best practices given its sensitivity to very small noise sources as well as the theoretical and empirical limitations of applying to severe brain injury patients processing and analytic algorithms that were developed for healthy volunteers [43, 44]. As research progresses, applicability and availability of any biomarker for the ICU context may change and guidance should be given as to which measures are highest yield if made broadly available. Functional MRI with motor imagery, PET, and transcranial magnetic stimulation with EEG co-registration offer particular scientific promise, but possess numerous challenges related to feasibility in real-world ICU settings.

### Benefits to the Patient, Family, and Clinicians

There are three distinct ways that biomarkers will benefit. For families, better and more accurate information is important. For clinicians, following progression of DOC in response to medications will give insight into the utility of the treatment. For researchers, correlating both better endotypic definition of DOC and disease progress over time with the site and magnitude of injury will be critical to make inferences about pathophysiology.

New biomarkers that accurately determine DOC endotypes could be used to guide patient treatments toward improved cognitive and functional outcomes. Families will receive more precise prognostic information and clinicians will better monitor patient trajectories in order to modify treatments. As has been mentioned previously, early withdrawal of life supporting interventions is a common cause of death in patients with DOC. Better information will improve the shared decision making between caretakers and families [6].

For development of treatment strategies, biomarkers will be critical to determine whether treatments make a difference. It is quite possible that hurdles in measuring improvement (or lack thereof) in DOC studies may represent the most important hurdle to finding therapies. It is possible that existing medications are useful in the treatment of patients with DOC, but the correct dose and timing is obscured by the inability to detect early improvement.

In the study of patients with DOC, biomarker development will also have an important role. Because human research limits the output variables available to correlate with pathology, improvements in biomarkers will allow researchers to make inferences about which pathology or network dysfunction is remediable.

### Next Steps to Biomarker Development

Given the plethora of existing potential biomarkers and the lack of clarity regarding actionable information for predicting and tracking recovery of consciousness in acutely injured patients, a two-pronged approach is needed. First, the most relevant anatomic and biochemical pathways should be identified that indicate the ability to emerge from coma and improve cognition. Prospective studies of highly characterized endotypes with clinical correlation and other candidate biomarkers will need to be undertaken. Early assessment at the time of injury and over a period of years will be necessary. Preliminary findings from these studies will drive sample size estimates to inform the breadth and scope ultimately needed for further study. In addition, changes in biomarker values with current clinical interventions and the relationship to coma recovery will refine mechanistic pathways.

To study the underpinnings of DOC, a mechanistic approach will suggest specific types of biomarkers that interrogate these pathways. Certain biomarkers that provide information regarding brain injury or outcome prediction but do not link directly to mechanistic pathways can be rejected as tangential to the primary project focus, while other biomarkers not yet in existence may need to be developed. Second, it is important to coalesce existing biomarker experience regarding neuroimaging, electrophysiology, serum and CSF studies, and physiology into a shared accessible dataset that allows cross-referencing of patients for exploration of mechanistic insights that may feed back into the first prong. Existing data, while disparate, is a powerful tool to utilize if regulatory, data sharing, and incentive issues can be worked out.

## Proof-of-Concept Clinical Trials to Promote Recovery of Consciousness in the ICU

ICU clinicians currently lack treatments proven to promote early recovery of consciousness during the first 28 days post-injury. Without knowing whether a patient can or will recover consciousness, families may withdraw life-sustaining therapy. This decision accounts for up to 70% of deaths in patients with DOC following TBI and over 40% of deaths in comatose patients with hypoxic-ischemic injury [5, 45].

To date, clinical trials of consciousness-promoting therapies in patients with DOC resulting from a structural cause have been performed almost entirely in the subacute-to-chronic stage of recovery [46]. The focus on late-stage interventions has likely resulted in missed early-intervention opportunities for restoring consciousness. Prior proof-of-concept trials have tested pharmacologic and electrophysiologic therapies for their effect on arousal and awareness (the two essential components of consciousness). Most pharmacologic trials have tested stimulant medications, particularly those that promote dopamine signaling within the brain, including amantadine [47], methylphenidate [12], levodopa [48, 49], bromocriptine [50], pramipexole [50], and apomorphine [51]. Zolpidem, a sleep aid, has also been tested in patients with subacute-to-chronic DOC because of its paradoxical awakening effect in a small subset of patients, which is hypothesized to be related to a change in background activity levels of thalamocortical circuits (mesocircuit) [52, 53]. Electrophysiological trials have tested deep brain stimulation [13], transcranial magnetic stimulation [54], transcranial direct current stimulation [15], low intensity focused ultrasound pulsation [55], and vagal nerve stimulation [56]. Yet, despite initial encouraging results from several proof-of-concept pharmacologic and electrophysiologic trials conducted in the subacute-to-chronic setting, there have been few studies of consciousness-promoting therapies in the acute ICU setting. In one example, an electroclinical response to a time-limited trial of an anti-seizure medication has been advanced as part of the criteria to diagnose indeterminate epileptiform activity constituting “probable nonconvulsive status epilepticus” (although a standardized approach to codifying an electroclinical response has yet to be validated) [57]. Regardless of etiology, the Curing Coma Campaign aims to advance the design and implementation of rigorous, proof-of-concept clinical trials in the ICU, with the ultimate goal of providing consciousness-promoting therapies to patients with acute DOC.

### Current State of Clinical Trial Development

Two major barriers have prevented the development of consciousness recovery therapy in the ICU: (1) patients are not rigorously endotyped prior to enrollment in clinical trials, and (2) therapeutic responses are not tested with appropriate measures of brain function in early-phase clinical trials. Instead of enrolling patients based on the pathophysiologic mechanism of coma, trials often enroll patients based on a behavioral measure such as the Glasgow Coma Scale (GCS) score or the Full Outline of UnResponsiveness (FOUR) Score, which grade brain injury on a continuum from mild to severe and focus on initial clinical severity rather than pathophysiology and trajectory [58, 59]. This approach is ineffective because coma is a highly heterogeneous and dynamic condition. Traumatic and hypoxic-ischemic coma, for example, are associated with variable combinations of axonal disconnections within brain networks essential for consciousness [60, 61]. Early-phase trials have also historically relied upon indirect serologic markers of brain injury or insensitive measures of disability [62]. Without biomarkers that directly measure brain function, fundamental questions about a therapy’s mechanisms of action cannot be answered in early-phase trials. These barriers have contributed to the 0% success rate for Phase 3 clinical trials conducted in patients with coma in the ICU. It is clear that a new, mechanistic approach to clinical trial design is needed [14, 62, 63].

### Approach to Solving the Problem

When designing new trials to promote recovery of consciousness in the ICU, two questions will need to be answered: (1) which substrates of consciousness are preserved, and (2) does the new therapy either engage these targets directly to restore consciousness or alternatively reverse an underlying illness temporarily disabling these targets? New tools are required to answer these questions. Specifically, tools are needed that identify preserved brain network connections in critically ill patients. Over the past decade, there have been rapid advances in the ability to map human brain networks that are essential for consciousness. These consciousness-supporting networks include the subcortical ascending arousal network, or reticular activating system, and cortical default mode network, both of which have recently been mapped in ICU patients with acute brain injuries [41, 61, 64]. There is an urgent need to develop network mapping techniques that can provide *repeated* bedside assessments during the acute phase in the ICU.

Early clinical trials may focus on biomarker validation and development in addition to intervention. The Curing Coma Campaign will support a multimodal approach in which a broad range of potential predictive biomarkers and pharmacodynamic biomarkers are tested in early-phase clinical trials. This approach has already demonstrated utility in predicting 6-month outcome in a cohort of patients with TBI [65].

### Benefits to the Patient, Family, and Clinicians

The clinical motivation for development of consciousness-promoting therapies in the ICU is that early recovery of consciousness could reduce the likelihood of premature withdrawal of life-sustaining therapy, facilitate self-expression, and increase access to rehabilitative care to accelerate the trajectory of recovery. Early recovery of consciousness will also decrease the risk of ICU-related complications (e.g., infection and deep vein thrombosis) that lead to higher morbidity and mortality rates.

### Next Steps to Developing Proof-of-Concept Clinical Trials

With recent studies showing that pharmacologic and electrophysiologic stimulation therapies can reactivate brain networks in selected patients, personalized connectome mapping tools and fluid biomarkers available at the point-of-care are urgently needed to identify patients who may benefit from these therapies. We envision that a principled, mechanistic approach to predicting and measuring responses to new therapies in the ICU could allow clinicians to provide targeted treatments that are personalized to each patient, ensuring that each patient is given the best possible chance to recover consciousness in the ICU and beyond.

To our knowledge, there are two ongoing or soon-to-be-launched clinical trials that incorporate the principles outlined above to engage targets in consciousness networks directly: Low Intensity Focus Ultrasound Pulsation (LIFUP; ClinicalTrials.gov NCT02522429), and Stimulant Therapy Targeted to Individualized Connectivity Maps to Promote ReACTivation of Consciousness (STIMPACT; ClinicalTrials.gov NCT03814356). Each trial aims to stimulate a preserved pathway within the connectome (the “wiring diagram” of the brain), though diagnostic tools to identify these pathways are still being refined and developed. With the support of the Curing Coma Campaign, these clinical trial efforts will leverage the expertise and experience of the neurocritical care and consciousness science communities.

## Conclusions

The “grand challenge” of Curing Coma is ambitious and could be seen by some as diffuse or unattainable. However, the issues faced by patients and the current gaps in prognostication and intervention necessitate that the multidisciplinary field of coma science and recovery come together and take a fresh, bold, and substantive new approach. The Curing Coma SAC and these initial proceedings describe three foundational pillars that represent a first step. Undoubtedly, this will require a large coordinated effort, for which analogies to the “moonshot” of the 1960s United States space program are inspirational [66, 67]. One of the additional conclusions of the SAC meeting is that its current membership, while useful to initiate the Curing Coma Campaign scientific agenda, has knowledge gaps that will necessitate contributions from many more members of the scientific, clinical, education, and implementation community. Thus, these proceedings should be seen as setting an initial framework whereby others can join, contribute, and drive the mission forward.

One interesting result of this review derives from common aspects identified across the three pillars and the intersection of the three toward the common goal of improving care of patients with coma and DOC. All three groups concluded that biomarkers that allow the correct identification and track progress of patients with impaired consciousness are critical to move the field forward. The biomarkers group further identified a number of different types of biomarkers including behavioral assessments, blood tests, physiological tests (such as EEG), and imaging tests that have relevance. In addition, while it may be acceptable to have complex tests used at the beginning of therapy to make initial endotypic diagnoses, tests that inform the ongoing progress of patients need to be interpretable at bedside. Further, biomarkers can be leveraged in different patient situations to make them more useful. For example, both resting EEG and passive and active perturbation tests can illicit more information about brain responses than either test alone.

Another important conclusion relates to the need for a rational system for endotyping patients with altered consciousness. Although many potential systems are possible, one based on the potential for treatment success may be the most rational. One construct could involve four basic endotypic categories: DOC endotype (1) without structural damage, (2) with structural damage that is amenable to replacement or bypass therapy, (3) with structural damage that is not amenable to replacement or bypass therapy, and (4) coma mimic endotype. A pragmatic approach such as this would focus attention on rapid identification of distinct trajectories of recovery which will inform more targeted treatment interventions. Furthermore, as new interventions become available, specific mechanisms of DOC may move across DOC endotypes (e.g., a previously untreatable type of coma becomes treatable).

Finally, the group charged with identifying real world strategies to test therapies concluded that the lack of appropriate biomarkers and limitations in endotyping individual patients make interventional trials difficult to construct and interpret. The development of *common data elements* for DOC is important for endotyping coma patients in the ICU and will be critical to the success of future clinical trials and biomarker development [68].

All of the groups identified specific barriers to studying patients with coma and impaired consciousness. A major barrier is that the specific nervous system circuits that gate consciousness are not fully understood and are difficult to image or test in real time, especially in acutely critically ill patients. Most current biomarkers and endotypes use the measurable, but imprecise, surrogate of whole-brain-impairment to make assessments about hard-to-measure specific consciousness circuit integrity. In the future, the ability to specifically investigate and target consciousness circuits will be a necessity. All three groups also identified the dearth of studies assessing complex mechanisms of consciousness impairment in the acute setting. Technological barriers to current biomarker use, especially imaging, in acute critically ill patients will need to be overcome. To date, most clinical trials focusing specifically on improving consciousness in impaired patients have been performed in the subacute and chronic settings. It may be that biomarkers and endotypes that are useful in later stages of illness may not be appropriate in the acute setting.

It is important to note that the challenges outlined in this paper focus on human investigations. The nature and complexity of consciousness makes it difficult to model in non-human animals. The limitations on mechanistic inferences in human clinical trials make it vitally important that researchers continue to develop non-human models that can test mechanistic aspects of consciousness and DOC. There is a critical need for animal and in silico models of brain activity that can be interrogated to investigate specific aspects of consciousness recovery. There is already a robust field of animal consciousness research that is beyond the scope of this document and is reviewed elsewhere [69].

In summary, these proceedings represent a first step toward framing the broad scientific challenges likely to be faced by the Curing Coma Campaign as it seeks to fundamentally improve the understanding and treatment of acute DOC. Reporting plans and progress as they unfold will be important in order to bring together the “curing coma community” across science, advocacy, education, and implementation. The NCS invites comments and collaboration as the Curing Coma Campaign proceeds and develops with the goal of awakening hope and improving the care of these most vulnerable patients.
